# Exploratory Cytokine and Bone-Marker Patterns in a Proteoglycan-Induced Spondyloarthritis Mouse Model: Th1/Th2 Strain Comparison and TLR2/3/4 Knockout Readouts

**DOI:** 10.3390/ijms27031337

**Published:** 2026-01-29

**Authors:** Johannes Dominikus Pallua, Michael Schirmer

**Affiliations:** 1 Department of Orthopaedics and Traumatology, Medical University of Innsbruck, 6020 Innsbruck, Austria; johannes.pallua@i-med.ac.at; 2 Department of Internal Medicine, Medical University of Innsbruck, 6020 Innsbruck, Austria

**Keywords:** spondyloarthritis, cytokines, toll-like receptor, bone markers, inflammation, innate immune system, mouse model

## Abstract

Validated biomarkers for clinical decision-making in spondyloarthritis (SpA) remain limited, and exploratory experimental studies may help prioritize candidate immune and bone-related readouts for future validation. In this pilot study, cytokine and bone-related biomarker profiles were analyzed in a proteoglycan-induced SpA model using Th1-prone C57BL/6J wild-type (WT) mice (non-immunized *n* = 8; immunized *n* = 16) and Th2-prone BALB/c WT mice (non-immunized *n* = 7; immunized *n* = 9), as well as immunized TLR2-knockout (KO) (*n* = 7), TLR3-KO (*n* = 8), and TLR4-KO (*n* = 3) strains on the C57BL/6J background. Serum cytokines were quantified longitudinally with a 26-plex immunoassay, and ELISA measured bone metabolism markers (DKK1, Wnt3a, Noggin). Cytokine analysis revealed distinct Th1/Th2 polarization: immunized Th1-prone C57BL/6J WT mice exhibited high Th1- and Th17-type cytokines (TNF-α, IFNγ, IL-12p70, IL-17A, and IL-22), whereas immunized Th2-prone BALB/c WT mice showed elevated Th2- and eosinophil-related cytokines (IL-4, IL-9, IL-13, IL-5, and RANTES). In TLR2-KO and TLR3-KO, Th1- and Th17-associated cytokines were markedly reduced, while Th2 cytokines were increased, confirming that TLR2 is essential for maintaining pro-inflammatory signaling. DKK-1 and Noggin levels were significantly higher in TLR2-KO mice, indicating altered terminal serum bone-marker profiles under immunized conditions. These findings indicate that Th1/Th2 immune backgrounds and TLR-associated contexts are associated with distinct cytokine patterns and differences in terminal bone markers in this experimental SpA model. Given the pilot design, small and imbalanced groups, missing non-immunized TLR-KO controls, and exploratory statistics without multiplicity adjustment, the results should be interpreted as hypothesis-generating and require confirmation in appropriately controlled, statistically powered studies incorporating longitudinal and structural endpoints, as the present findings are exploratory and not directly translatable to clinical biomarker use or therapeutic decision-making.

## 1. Introduction

From a clinical perspective, spondyloarthritis (SpA) is a chronic inflammatory disease that affects both axial and peripheral joints. SpA encompasses a spectrum of related disorders, including undifferentiated SpA, reactive arthritis, and SpA associated with psoriatic disease, uveitis, or inflammatory bowel disease. All these subtypes can potentially progress into ankylosing spondylitis (AS), the most severe form of axial SpA. With reported prevalences ranging from 0.20% to 1.61%, SpA is the most common chronic inflammatory rheumatic disease [1]. Despite substantial advances in the field, the pathogenesis of SpA remains incompletely elucidated. Polymorphisms at different stages of the disease may play a role and partly explain the great variety of disease courses over the years. The individual relation between Th1- and Th2-responsiveness to stimuli may alter the disease course. Thus, a complex interplay between genetic, environmental, and cellular components, including inflammatory cytokines and chemokines, underlies disease development [2].

The main pathogenetic factors of SpA are certainly genetic, especially the human leukocyte antigen-B27 (HLA-B27), encoded on chromosome 6, which has the strongest association with SpA. Yet, the precise role of HLA-B27, a member of the major histocompatibility complex (MHC) class I molecules, in the disease process remains incompletely understood. Proposed mechanisms include alterations in antigen presentation to CD8+ T cells, recognition of misfolded HLA-B27 heavy chains by immune cells, and the induction of the unfolded protein response (UPR) due to endoplasmic reticulum (ER) stress. UPR activation can lead to interleukin-23 (IL-23) secretion, promoting inflammatory pathways implicated in SpA pathogenesis [3]. The IL-23/IL-17 axis is now recognized as a critical driver of SpA, and IL-17-producing cells play a pivotal role in mediating inflammation and tissue remodeling [4]. Moreover, HLA-B27 may directly stimulate pro-inflammatory CD4+CD28- T cells via natural killer (NK) cell receptors, further amplifying immune responses [5]. Beyond HLA-B27, other non-HLA genetic variants, such as those in ER aminopeptidase (ERAP) 1 and 2 and the IL-23 receptor (IL-23R), contribute to SpA susceptibility. ERAP1 and ERAP2 modulate peptide trimming for MHC class I binding and, in combination with HLA-B27, may shape the antigenic landscape in SpA [6]. Multiple cytokines have been implicated in SpA, with tumor necrosis factor-α (TNF-α), IL-17A, and IL-12/IL-23 representing key therapeutic targets [7,8,9]. Polymorphisms in IL-23R are associated with an increased risk of AS and psoriatic arthritis [10], and IL-23 is a critical factor in the development of enthesitis [10]. IL-23 receptor activation on resident enthesis T cells induces the secretion of cytokines such as IL-17, IL-22, IL-6, and CXCL1/Gro-α [10]. Of note, IL-22 promotes osteogenesis in human mesenchymal stem cells, linking cytokine-mediated inflammation to pathological bone formation in SpA [11]. Historically, SpA was considered a Th1-mediated disease, driven by interferon-γ (IFNγ) and IL-2 [12,13]. However, recent insights have shifted the focus to Th17 cells and IL-17, although hybrid Th17/Th1 phenotypes are also described [12]. Significantly, Th17 cells can be activated by IL-23 through IL-23R signaling [12], underlining the relevance of this pathway in SpA pathophysiology. The Wnt signalling pathway is a key regulator of bone formation, acting via stabilization of β-catenin and promoting osteoblast differentiation [14]. Counteracting this pathway, Dickkopf proteins, particularly Dickkopf-1 (DKK1), serve as potent Wnt inhibitors and thus regulate bone homeostasis [14,15]. In parallel, bone morphogenetic proteins (BMPs), members of the transforming growth factor-β (TGF-β) family, contribute to endochondral bone formation and are upregulated during ankylosis in animal models of SpA. Noggin, a known antagonist of BMPs, modulates these osteogenic signals and may influence pathological bone remodeling [16].

Innate immunity, as represented by pattern recognition receptors such as toll-like receptors (TLRs), also plays a critical role in SpA. TLRs detect pathogen-associated molecular patterns and initiate inflammatory signaling cascades. Each TLR subtype recognizes distinct ligands: TLR4 binds lipopolysaccharides of Gram-negative bacteria, TLR2 recognizes lipoteichoic acid and peptidoglycan from Gram-positive bacteria, and TLR3 detects viral double-stranded RNA. Beyond infections, TLRs also mediate sterile inflammatory responses relevant to autoimmune diseases like SpA [17]. Previous research demonstrated TLR expression on pro-inflammatory T-helper cells in AS patients [18]. This observation suggests a role for microbial triggers or TLR-mediated pathways in modulating disease activity and shaping cytokine profiles.

Biomarkers are defined as “a characteristic that is objectively measured and evaluated as an indicator of normal biological processes, pathogenic processes, or pharmacologic responses to a therapeutic intervention”. To be clinically applicable, biomarkers require validation regarding their analytical accuracy, clinical relevance, and utility for improving patient outcomes [19]. Ideal biomarkers are sensitive, specific, reproducible, and preferably obtainable via non-invasive procedures. Biomarkers can be molecular, imaging-based, or clinical. Molecular biomarkers, such as HLA-B27, ERAP1, and IL-23R, are measurable in biological fluids or tissues; erythrocyte sedimentation rate and C-reactive protein levels are nonspecific. Clinical biomarkers include physical signs and symptoms, and may complement diagnostic and monitoring tools [20]. At present, only imaging modalities such as sonography, magnetic resonance imaging, and computed tomography provide objective assessments of disease activity and structural damage but are too late to detect structural changes once they have occurred.

The increasing availability of molecular biomarkers, notably cytokines, facilitates their use in clinical and research settings. These cytokine networks involve various immune cell types, including dendritic cells and macrophages (producers of IL-23), innate immune cells (sources of IL-17), and adaptive T cells, particularly Th17 subsets [21,22]. To investigate the complex immunopathogenesis of SpA, diverse animal models have been established [23]. Among these, the BALB/c mouse model, developed by T. Glant through immunization with fetal human cartilage proteoglycan and Freund’s adjuvant, mimics features of AS [24]. These models, utilized in both short-term and long-term experimental settings, enable the study of disease mechanisms and biomarker discovery. C57BL/6 (Th1-prone) and BALB/c (Th2-prone) mice offer contrasting immunological backgrounds, crucial for exploring T-helper cell-related cytokine profiles in SpA. Since human SpA is considered a Th1-driven condition [13], Th1-prone mice are often preferred for translational research, while Th2-prone models such as BALB/c serve as valuable comparators for dissecting immune deviations. In addition to the well-established Th17/Th1-driven immune responses in SpA, eosinophil-associated type 2 immune signatures warrant consideration when interpreting cytokine patterns across distinct immune backgrounds. Markers of eosinophil activation have been investigated in ankylosing spondylitis, including elevated circulating eosinophil cationic protein despite unchanged eosinophil counts, and blood eosinophil parameters may be influenced by inflammatory state and anti-TNF therapy [25,26]. Eosinophils can modulate inflammatory tissue responses via the release of cytotoxic granule proteins and lipid mediators and by shaping cytokine networks through IL-5 and the IL-4/IL-13 axis, with functional links to innate immune signaling [27,28,29]. Therefore, including eosinophil-related readouts (e.g., IL-5 and related type 2 cytokines/chemokines) provides additional resolution for subgroup-specific immune patterns and supports a more nuanced interpretation of Th1/Th2 polarization in experimental SpA models. Despite significant therapeutic advances, there remains a strong need for reliable molecular biomarkers in SpA. In this context, capturing eosinophil-associated type 2 mediators (e.g., IL-5 and related chemokines) alongside Th1/Th17 markers may help resolve subgroup-specific inflammatory patterns and improve the interpretability of cytokine-based biomarker profiles. To date, only the erythrocyte sedimentation rate (ESR) and the C-reactive protein (CRP) are routinely used, yet both lack disease specificity and sensitivity. Numerous potential biomarkers have been proposed across individual studies, but an integrated analysis encompassing Th1-, Th2-, and Th17-associated mediators has not been performed. To address this gap, the present pilot study evaluates cytokine and bone-related biomarker patterns in Th1- and Th2-prone murine models of experimental SpA, as well as in TLR knockout (KO) variants.

This pilot study aimed to systematically investigate cytokine and bone-related biomarker profiles in experimental spondyloarthritis using Th1-prone (C57BL/6J) and Th2-prone (BALB/c) mouse strains, as well as TLR2-, TLR3-, and TLR4-KO models on the C57BL/6J background. By combining multiplex cytokine profiling with enzyme-linked immunosorbent assay (ELISA)-based quantification of bone metabolism markers (DKK1, Wnt3a, Noggin), this work sought to determine how Th1/Th2 polarization and TLR signaling influence inflammatory and osteogenic pathways. The study aims to characterize cytokine and bone-marker patterns across immune backgrounds and TLR-KO conditions as a hypothesis-generating step toward candidate biomarkers requiring independent validation.

This pilot study reports cytokine profiles in Th1-prone (C57BL/6) and Th2-prone (BALB/c) mouse models of SpA, in wild-type groups studied under non-immunized and immunized conditions, and in TLR2/3/4-KO groups studied only under immunized conditions. By evaluating these profiles, the study explores candidate cytokine and bone-marker patterns associated with immune background and TLR signaling, contributing to a better understanding of innate immunopathogenic mechanisms and informing the design of future, appropriately controlled validation studies.

## 2. Results

Group sizes were as follows: Th1-prone C57BL/6J WT non-immunized (*n* = 8) and immunized (*n* = 16); Th2-prone BALB/c WT non-immunized (*n* = 7) and immunized (*n* = 9); immunized TLR2-KO (*n* = 7), TLR3-KO (*n* = 8), and TLR4-KO (*n* = 3) on the C57BL/6J background. Unless otherwise stated, these *n* values apply to the corresponding group comparisons. Where individual time points contain missing measurements (e.g., due to limited serum volume), the effective *n* is indicated in the respective figure panel/legend. Because the TLR4-KO group is tiny (*n* = 3), all TLR4-related results are presented descriptively only. Planned non-immunized TLR-KO control cohorts were not included because of early animal loss before longitudinal sampling; therefore, all KO comparisons refer to immunized cohorts only.

Radar plots are presented to visualize longitudinal patterns across time points and endpoints; inferential statements are restricted to the statistical comparisons described in the Methods. Cytokine and biomarker profiles were analyzed using an established proteoglycan-induced murine SpA model as described in the Section 4, focusing on differences between Th1- and Th2-prone mice and on the contribution of TLR2-, TLR3-, and TLR4-pathways in the Th1-prone background [30].

In addition to visualization, longitudinal kinetics were assessed using repeated-measures models including a group × time interaction term. Interaction *p*-values (and family-wise BH–FDR *q*-values across cytokines per comparison) and AUC-based kinetic effect sizes are reported in the Supplementary Materials (Table S2). These longitudinal analyses were used to support statements on kinetic differences, whereas the radar plots are retained for descriptive visualization.

Given the exploratory design and multiple comparisons, interpretation emphasizes effects that remain consistent after family-wise BH-FDR correction (*q*-values); complete *p*/*q* tables are provided in the Supplementary Materials (Table S1). For clarity, the Results are organized by comparison frame: (i) strain effects comparing proteoglycan-immunized C57-PG vs. BALB-PG (with non-immunized strain controls), and (ii) TLR effects comparing immunized C57-PG vs. immunized C57-TLRxKO-PG within the C57BL/6J background.

Central figures focus on prespecified key comparisons (immunized vs. non-immunized within strain; immunized C57BL/6J vs. immunized BALB/c; immunized TLR2-KO vs. immunized WT; immunized TLR3-KO vs. immunized WT); complete comparison matrices with *p* and *q* values are reported in Supplementary Tables S1–S3. Radar plots are retained as overview visualizations; however, quantitative comparisons are emphasized using key-cytokine line plots and AUC summaries in the main text.

### 2.1. Strain Effect (Immunized): C57-PG vs. BALB-PG (With Non-Immunized Controls)—Th1/Th2/Th17 Cytokines

**Figure 1:**
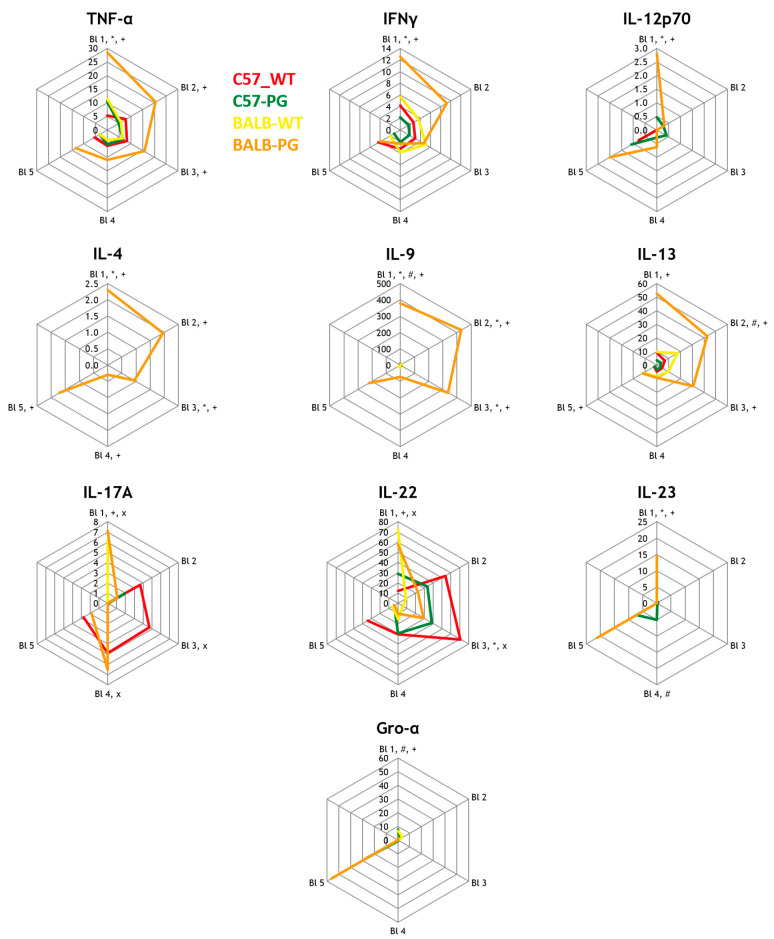
Strain effect (immune background): C57-PG vs. BALB-PG with non-immunized strain controls. Longitudinal serum cytokine concentrations (26-plex) are shown as radar plots of median values across bleeding time points B1–B5 (angular axis) with concentration (pg/mL) on the radial axis. Groups are displayed in a consistent order and color scheme: C57-WT (red; *n* = 8), C57-PG (green; *n* = 16), BALB-WT (pink; *n* = 7), BALB-PG (orange; *n* = 9). Predefined pairwise comparisons at each time point were tested using Mann–Whitney U tests; symbols next to the time-point label indicate which comparison(s) were tested and reached nominal significance at that time point, using the following key: * C57-PG vs. C57-WT; + BALB-PG vs. BALB-WT; # C57-PG vs. BALB-PG; × C57-WT vs. BALB-WT. Complete unadjusted *p*-values and BH–FDR-adjusted *q*-values (family-wise) for all cytokines/time points are provided in the Supplementary Materials (Table S1). Abbreviations: TNF-α, tumor necrosis factor alpha; IFNγ, interferon gamma; IL, interleukin; Gro-α/CXCL1, growth-regulated oncogene alpha/chemokine (C-X-C motif) ligand 1.

As shown in Figure 1, long-term analysis of markers of the adaptive immune response revealed a strong Th1 and Th17 bias in C57BL/6J mice compared to BALB/c mice following proteoglycan-induced SpA. Immunized C57BL/6J mice showed markedly elevated levels of TNF-α, IFNγ, IL-12p70, and IL-17A, along with increased IL-22 and IL-23 levels, confirming the induction of a pathogenic inflammatory axis typical of human SpA [4,7,10,12,13]. In contrast, Th2-related cytokines, such as IL-4, IL-9, and IL-13, were significantly elevated in BALB/c mice, reflecting their Th2-prone immune profile [12,13]. C57BL/6J mice exhibited minimal Th2 cytokine production, underscoring polarization toward pro-inflammatory and bone-relevant cytokines such as IL-17A and IL-22 [4,11]. These findings demonstrate strain-specific cytokine profiles: C57BL/6J mice exhibit a Th1/Th17-skewed profile, whereas BALB/c mice show elevated Th2 cytokine levels. Baseline (B1) sensitivity analyses (Δ from B1 and/or baseline-adjusted models where feasible) yielded qualitatively consistent patterns, suggesting that the observed immunized-group differences are not solely explained by baseline intergroup variation. Repeated-measures modeling supported kinetic differences (group × time interaction) for selected key cytokines in this comparison; complete interaction statistics and AUC-based effect sizes are provided in the Supplementary Materials (Table S2).

### 2.2. Strain Effect (C57-PG vs. BALB-PG) and TLR Effect (C57-PG vs. C57-TLRxKO-PG) on Eosinophil-Associated Cytokines

**Figure 2:**
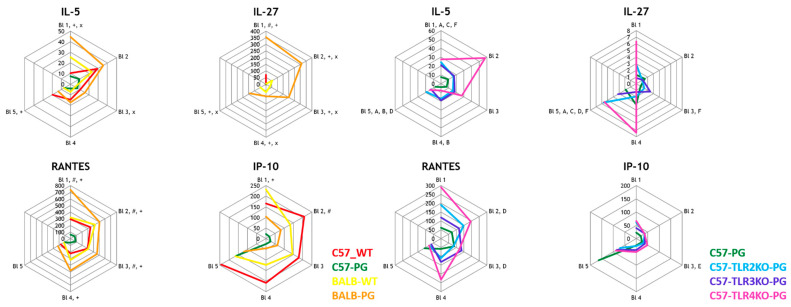
Type 2 cytokine module (IL-4, IL-5, IL-9, and IL-13) and IFN-inducible chemokines (IP-10/CXCL10)—strain effect and TLR effect under immunized conditions. Radar plots show median longitudinal concentrations across bleeding time points B1–B5. IL-4, IL-5, IL-9, and IL-13 are presented as a type 2/eosinophil-associated cytokine module, whereas IP-10 (CXCL10) is presented as an IFN-inducible chemokine; IL-27 is discussed as an immunoregulatory cytokine rather than an eosinophil marker [31,32,33,34]. Left panel (strain effect): groups and colors as in Figure 1 (C57-WT grey *n* = 8; C57-PG green *n* = 16; BALB-WT yellow *n* = 7; BALB-PG orange *n* = 9), with the same symbol key for predefined comparisons: + BALB-PG vs. BALB-WT; # C57-PG vs. BALB-PG; × C57-WT vs. BALB-WT. Right panel (TLR effect; immunized C57 background): C57-PG (black; *n* = 16) compared with C57-TLR2KO-PG (cyan/blue; *n* = 7), C57-TLR3KO-PG (purple; *n* = 8), and C57-TLR4KO-PG (red; *n* = 3). Letters at each time point indicate which pairwise comparisons among these four immunized C57 groups were tested and reached nominal significance. All tests used Mann–Whitney U; complete *p*-values and BH–FDR *q*-values (family-wise) are provided in the Supplementary Table S1. TLR4-KO (*n* = 3) is underpowered and is interpreted descriptively. Abbreviations: RANTES/CCL5, regulated upon activation, normal T cell expressed and secreted; IP-10/CXCL10, interferon gamma-induced protein 10; IL, interleukin.

In this section, we report a Type 2 cytokine module (IL-4, IL-5, IL-9, and IL-13) and distinguish it from IFN-inducible chemokines (IP-10/CXCL10). IL-27 is discussed separately as an immunoregulatory cytokine that can support type 1–associated programs and may induce CXCL10 in specific contexts, and is therefore not treated as an eosinophil-associated readout [12,13,31,32,33,34]. As shown in Figure 2, BALB/c mice displayed higher baseline levels of these mediators than C57BL/6J wild-type mice, and immunization increased these cytokines in C57BL/6J mice, whereas the corresponding responses were reduced in TLR2-, TLR3-, and TLR4-deficient groups. However, these cytokines were also induced in C57BL/6J wild-type mice following immunization but were significantly reduced in TLR2-, TLR3-, and TLR4-KO mice, demonstrating that innate receptors contribute to amplifying eosinophil-related cytokine production [17,18]. The findings suggest that TLR signaling supports Th1 and Th17 responses and modulates eosinophil cytokine pathways that may contribute to tissue inflammation in SpA [17,18]. This functional grouping follows established immunological definitions of type 2 cytokines (IL-4/IL-5/IL-9/IL-13) and the IFN-γ–inducible chemokine CXCL10/IP-10 [31,32,33,35]. The group differences in eosinophil-related cytokines are shown in Figure 2.

### 2.3. TLR Effect (Immunized C57 Background): C57-PG vs. C57-TLR2/3/4KO-PG—Adaptive Cytokines

**Figure 3:**
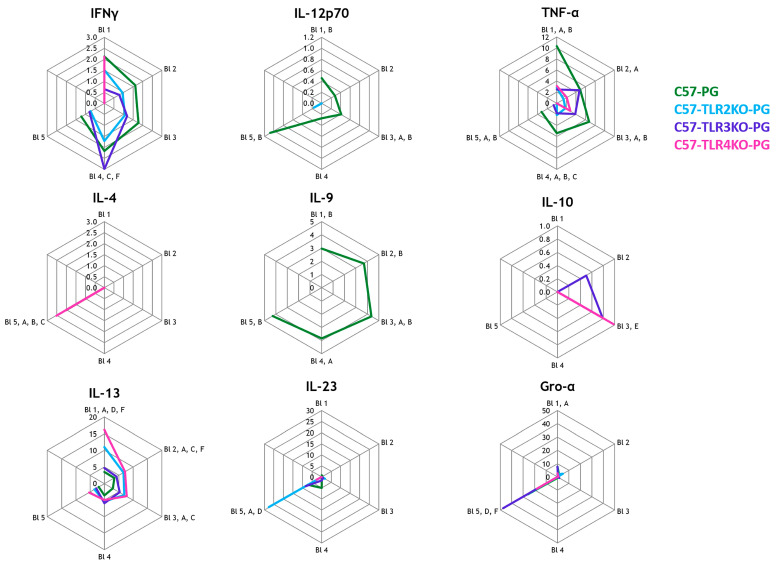
TLR effect (immunized C57 background): C57-PG vs. C57-TLR2/3/4KO-PG. Radar plots show median longitudinal cytokine concentrations across B1–B5 in proteoglycan-immunized C57BL/6J WT (C57-PG; green; *n* = 16) and immunized knockout cohorts TLR2KO-PG (cyan/blue; *n* = 7), TLR3KO-PG (purple; *n* = 8), TLR4KO-PG (pink; *n* = 3). Letters at each time point indicate pairwise comparisons among the four immunized C57 groups that reached nominal significance: A C57-PG vs. TLR2KO-PG; B C57-PG vs. TLR3KO-PG; C C57-PG vs. TLR4KO-PG; D TLR2KO-PG vs. TLR3KO-PG; E TLR2KO-PG vs. TLR4KO-PG; F TLR3KO-PG vs. TLR4KO-PG (Mann–Whitney U tests). Complete *p* and BH–FDR *q*-values (family-wise) are provided in the Supplementary Table S1. TLR4-KO (*n* = 3) is interpreted descriptively. Abbreviations: TLR, toll-like receptor; KO, knockout; IL, interleukin.

Because non-immunized KO controls were not available and KO cohorts were restricted to the C57BL/6J background, all TLR comparisons should be interpreted as exploratory differences under immunized conditions rather than baseline genotype effects. Cytokine profiles in TLR2-, TLR3-, and TLR4-KO mice showed quantitative differences in Th1-, Th2-, and Th17-associated mediators compared with wild-type animals. Comparisons involving TLR2-KO (*n* = 7) and TLR3-KO (*n* = 8) were interpreted as exploratory; TLR4-KO (*n* = 3) was not used for inferential/statistical interpretation and is shown for descriptive context only. Th1 cytokines (TNF-α, IFNγ, and IL-12p70) were significantly lower in TLR2-deficient mice. A reduction in IFNγ and IL-23 was also observed in TLR3-deficient mice. Conversely, Th2 cytokines IL-4 and IL-13 were higher in TLR2- and TLR3-deficient mice than in wild-type controls. IL-17A, IL-22, and Gro-α were reduced in both TLR2 and TLR3-KO animals (Figure 3). Longitudinal group × time interaction testing for KO–WT comparisons is summarized in the Supplementary Materials (Table S2); given the minimal TLR4-KO group size (*n* = 3), TLR4-related kinetics are presented descriptively only.

### 2.4. Strain Effect (C57-PG vs. BALB-PG) and TLR Effect (C57-PG vs. C57-TLRxKO-PG) on Macrophage-Associated Cytokines

**Figure 4:**
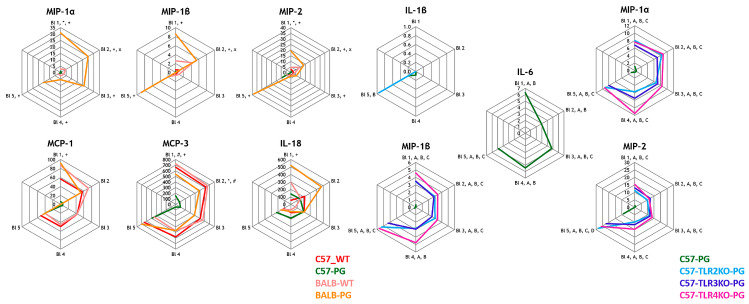
Macrophage-associated cytokines/chemokines—(**left**) strain effect and (**right**) TLR effect under immunized conditions. Radar plots show median longitudinal concentrations across B1–B5. Left panel (strain effect): groups/colors as in Figure 1 (C57-WT red *n* = 8; C57-PG green *n* = 16; BALB-WT pink *n* = 7; BALB-PG orange *n* = 9). Predefined comparison symbol key: * C57-PG vs. C57-WT; + BALB-PG vs. BALB-WT; # C57-PG vs. BALB-PG; × C57-WT vs. BALB-WT. Right panel (TLR effect; immunized C57 background): C57-PG (green *n* = 16) vs. TLR2KO-PG (cyan/blue *n* = 7), TLR3KO-PG (purple *n* = 8), TLR4KO-PG (pink *n* = 3). A–D as defined in Figure 2 (Mann–Whitney U tests). Complete *p* and BH–FDR *q*-values (family-wise) are provided in the Supplementary Table S1. TLR4-KO (*n* = 3) is interpreted descriptively. Abbreviations: MCP-1/CCL2, monocyte chemoattractant protein 1; MCP-3/CCL7, monocyte chemoattractant protein 3; MIP-1α/CCL3, macrophage inflammatory protein 1 alpha; MIP-1β/CCL4, macrophage inflammatory protein 1 beta; MIP-2/CXCL2, macrophage inflammatory protein 2; IL, interleukin.

Macrophage-associated cytokines including IL-1β, IL-6, MIP-1α, MIP-1β, MIP-2, MCP-1, and MCP-3 were elevated in immunized C57BL/6J mice compared with BALB/c mice. These cytokines were lower in TLR2- and TLR3-deficient groups than in wild-type animals. IL-6 showed the largest difference between TLR2-KO and WT mice. Overall, the data show strain- and TLR-dependent variation in macrophage-related cytokines (Figure 4).

### 2.5. TLR Effect (Terminal Serum Bone Markers): C57-PG vs. C57-TLR2/3/4KO-PG

**Figure 5:**
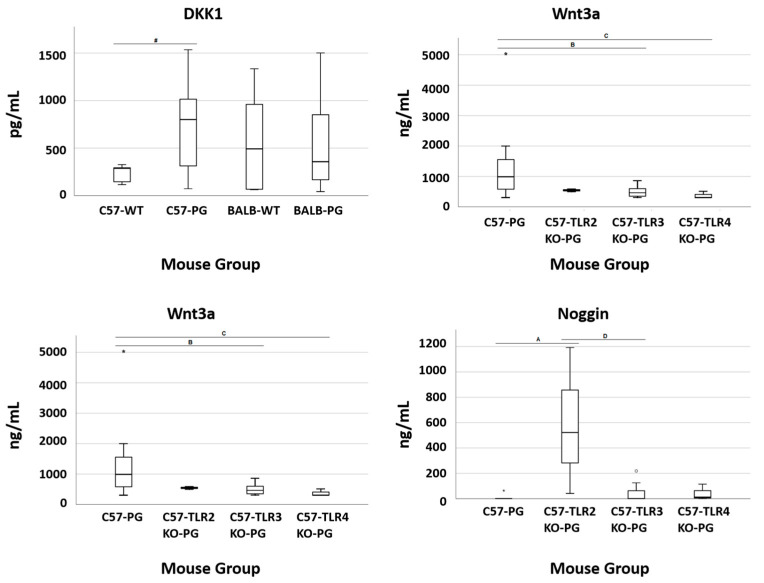
TLR effect on terminal serum bone-marker levels (final time point): C57-PG vs. C57-TLR2/3/4KO-PG. Boxplots show terminal serum concentrations of DKK1 (pg/mL), Wnt3a (ng/mL), and Noggin (ng/mL) in proteoglycan-immunized C57BL/6J WT (C57-PG; *n* = 16) and immunized knockout cohorts TLR2KO-PG (*n* = 7), TLR3KO-PG (*n* = 8), and TLR4KO-PG (*n* = 3). Boxes represent median and interquartile range; whiskers indicate data spread as defined in the Methods. Pairwise Mann–Whitney U tests were performed; complete *p* and BH–FDR *q*-values (family-wise across bone markers per comparison) are provided in the Supplementary Table S1. Given TLR4-KO (*n* = 3), TLR4-related findings are descriptive. Letters (A–D) above the brackets denote statistically significant pairwise group differences identified by the Mann–Whitney U tests with BH–FDR correction; exact *p* and *q* values for each comparison are reported in the Supplementary Table S1. Symbols (*, #, ○) shown in the figure indicate additional annotations of statistical comparisons/significance as marked in the plot; their interpretation is based on the same Mann–Whitney U testing with BH–FDR correction, with exact *p* and *q* values provided in the Supplementary Table S1. Abbreviations: DKK1, Dickkopf-related protein 1; Wnt3a, Wingless-type MMTV integration site family member 3A.

Bone metabolism-related markers were quantified at the terminal time point. DKK1 concentrations were higher in TLR2-KO mice than in wild-type mice. Noggin concentrations were also higher in TLR2-KO mice, whereas Wnt3a concentrations were higher in wild-type mice compared with TLR-deficient groups. Group distributions and statistical comparisons are shown in Figure 5.

## 3. Discussion

The results obtained across Th1- and Th2-prone strains and TLR-deficient models provide quantitative evidence of distinct cytokine and bone marker expression patterns. These observations are interpreted below in the context of known SpA pathomechanisms.

Moreover, after immunization, data from Th1-prone wild-type C57 mice were compared with those from KO C57 mice for TLR2, TLR3, and TLR4. The description of differentially expressed biomarkers has not been reported before and is challenging to perform in small studies. Some murine model biomarker studies provided interesting data but were not identified in the systematic literature review as possibly relevant for monitoring disease activity or radiographic progression. The most prominent finding of this work is a stronger cytokine expression after immunization in Th2-prone BALB/c mice than in Th1-prone C57 mice. This finding is especially evident in the early phase after immunization for Th1- and Th2-type cytokines. So far, SpA has been more often described as a Th1-type disease [13], and only a few studies have characterized SpA as a disease with an impaired Th1 cytokine profile [36], supporting the alternative concept of SpA as a Th2-prone disease.

Within the proteoglycan-induced SpA mouse model and the examined time window, the observed cytokine patterns indicate a relative enrichment of type 2–associated serum cytokines in Th2-prone BALB/c mice, in contrast to the Th1/Th17-skewed profile observed in Th1-prone C57BL/6J mice. These findings are model- and context-specific and do not imply that SpA is a Th2-prone disease per se.

This work clearly shows that immunized Th1-prone C57BL/6J WT mice exhibit higher Th1/Th17-type cytokine levels than immunized Th2-prone BALB/c WT mice, particularly in the early phase after immunization, whereas Th2/eosinophil-associated cytokines predominate in the Th2-prone BALB/c background. Th2-type cytokines, however, normalize during the disease course, then remain elevated or become elevated again at the end of the study. As expected, the Th2-type cytokines remain elevated in BALBcimm for a more extended period when compared to the Th1-type cytokines.

In line with our Results, TNF-α is higher in immunized Th1-prone C57BL/6J WT mice than in immunized Th2-prone BALB/c WT mice; accordingly, biologics targeting TNF-α have shown significant clinical efficacy in SpA patients [37]. The induction of UPR-related inflammatory signaling has been linked to TNF-α, IFNγ, and the IL-17/IL-23 axis [38], which is consistent with the Th1/Th17-skewed cytokine pattern observed in the immunized Th1-prone background. In our dataset, IL-17A and IL-22 were higher in immunized Th1-prone C57BL/6J WT mice, whereas Th2/eosinophil-associated cytokines predominated in immunized Th2-prone BALB/c WT mice. Gro-α may be more pronounced in immunized Th1-prone C57BL/6J WT mice. Importantly, these observations must be interpreted in the context of the well-established IL-23/IL-17 axis paradigm, which is supported by extensive genetic, experimental, and clinical evidence in SpA. The present findings do not contradict this framework; instead, they reflect serum cytokine patterns measured at defined time points in a specific experimental model. Type 2–associated cytokines detected in the circulation may represent systemic immune modulation, counter-regulatory responses, or strain-dependent immune polarization, rather than primary drivers of joint or entheseal pathology. Moreover, immune dominance in SpA is known to be time-dependent, with early inflammatory phases potentially differing from later or chronic stages.

The finding that IL-23 is higher after immunization in both BALB/c and C57_WT is consistent with a previous report by Mei Y. et al., which showed that serum IL-23 levels were higher in AS patients than in controls [39]. Also, treatment approaches specific to Th17-type cytokines are already in clinical use.

Data on the role of other cytokine groups are rare in the literature. For example, little is known about the role of eosinophils in the pathogenesis of SpA, whereas this work shows that several eosinophil-associated cytokines were lower in immunized Th1-prone C57BL/6J WT mice compared with immunized Th2-prone BALB/c WT mice and non-immunized Th1-prone C57BL/6J WT controls.

Concerning macrophage (MΦ)-related cytokines, some are higher only in early disease, whereas others, such as MIP-1α, MIP-1β, and MIP-2, are also elevated at the end of the disease course. MΦ -related cytokines appear more critical in the Th2-prone than in the Th1-prone mouse model. Regarding MIP-1α, our results align with the report by Akbulut H. et al., who showed higher MIP-1α in AS patients than in the control group [40]. They also found that anti-TNF therapies reduced MIP-1α levels in the AS group [40], suggesting that MIP-1α may serve as a potential biomarker of therapy response, at least in the early phase of the disease.

Interestingly, IL-6 data do not differ between BALB/c, BALBcimm, C57_WT, and C57_WTimm, suggesting that IL-6 is not relevant to the pathogenesis of SpA. Indeed, blockade of the IL-6 receptor with a monoclonal antibody (Tocilizumab, RoActemra^®^) was ineffective in patients with axial SpA and inconsistently effective in those with peripheral SpA [41]. Unfortunately, data on biomarkers directly involved in bone modeling (Wnt3a, a promoter of bone formation, and DKK1 and Noggin, antagonists of bone formation) were available only for the last bleeding. At this time, DKK1 was higher in Th1-prone C57_WTimm than in C57_WT mice, consistent with human studies showing higher DKK1 levels in the sera of SpA patients than in controls [42]. Interestingly, DKK1 is not elevated in Th2-prone BALB/c mice after immunization. However, in human studies, functional DKK1 levels were higher in AS patients without syndesmophytes than in those with syndesmophytes, supporting the concept that DKK1-induced reduction of Wnt signalling suppresses new bone formation [15]. The functional DKK1 level could therefore be used as a biomarker of structural progression in AS [15]. Concerning the role of TLRs in the pathogenesis of SpA, the use of TLR-KO mice was limited to the immunized Th1-prone C57 models. Interestingly, after immunization, several biomarkers are lower in the TLR-KO mice than in the WT mice at different time points (TNF-α, IFNγ, IL-12p70, IL-9, Gro-α, IL-1β, IL-6, and Wnt3a), whereas other biomarkers are increased (IL-4, IL-13, IL-5, IL-27, IL-23, MIP-1α, MIP-1β, MIP-2, DKK1, and Noggin). Keiko Ozato et al. reported that TLR signaling leads to stimulation of IL-1, TNF-α, IL-12, IL-6, and IL-10 and that TLR4-KO mice did not express TNF-α, IL-6, and IL-12 after stimulation with lipopolysaccharides [43]. Others reported that, in mice, E. coli lipopolysaccharides via TLR4 induced a Th1-like response with little or no IL-4, IL-13, or IL-5, whereas TLR4-independent activation induced Th2-type cytokines [44]. The final influence of TLRs on the differential expression of the biomarkers remains open.

As mentioned above, this work has several significant limitations. The interpretation of TLR-related effects is constrained by the absence of non-immunized TLR2/3/4-KO cohorts on the C57BL/6J background (excluded due to early mortality) and by the lack of corresponding TLR-KO cohorts on the Th2-prone BALB/c background. Consequently, TLR-associated differences observed here cannot be disentangled from immune-background effects or immunization-dependent responses, and should be interpreted strictly as comparisons under immunized conditions within a C57BL/6J background. First, the groups of mice were small, with a lack of non-immunized TLR-KO mice on C57 background and immunized and non-immunized TLR-KO mice on the BALB/c background. Also, data on bone formation markers (DKK1, Wnt3a, and Noggin) were available only for the last bleeding, as these biomarkers were measured by ELISA, which requires more blood to test all three markers. Thus, statistical evaluations remain preliminary, and future work with larger groups of mice is necessary to achieve conclusions on the different hypotheses generated by this work. Accordingly, the present study should be regarded as a pilot investigation. Accordingly, the ELISA findings should be interpreted strictly as terminal serum bone marker differences, not as evidence of bone remodeling, osteogenesis, or structural progression. Structural endpoints were not collected in the present cytokine-focused experiment. As a complementary context, the structural bone microarchitecture of the sacrum and lumbar vertebrae was assessed by blinded micro-CT in a related proteoglycan-induced SpA cohort (BALB/c WT, TLR2-KO, TLR4-KO; immunized and non-immunized) [45], but these imaging data are not directly linked to the present longitudinal cytokine dataset.

Although differences in cytokine patterns and terminal bone-marker levels were observed across strains and TLR-deficient groups, the present work is a hypothesis-generating pilot study and does not permit mechanistic attribution or translational inference. The study design includes small and imbalanced groups. It lacks critical controls, most importantly, non-immunized TLR2/3/4 knockout groups on the C57BL/6J background and corresponding TLR knockout groups on the BALB/c background, which limits the separation of immune-background effects from TLR-associated effects. In addition, multiple endpoints at several time points were examined with extensive pairwise comparisons, without a multiplicity-adjusted confirmatory framework; therefore, *p*-values should be interpreted cautiously as exploratory. Bone-related serum markers (DKK1, Wnt3a, and Noggin) were assessed only at the terminal time point and without structural endpoints within this dataset (e.g., imaging or histology), precluding conclusions regarding remodeling dynamics or structural progression. Finally, no clinical validation was performed (e.g., correlations with disease activity, imaging progression, reproducibility, thresholds, or independent cohorts). In particular, no standardized arthritis scoring/activity indices, functional readouts, or semiquantitative histology/imaging metrics of inflammation severity were collected for correlation with cytokine trajectories. Thus, the term ‘biomarker’ is used here in the sense of candidate experimental readouts rather than clinically actionable markers. Accordingly, statements implying therapeutic targeting of TLR2/TLR3 or clinically relevant ‘biomarker profiles’ have been removed; instead, the reported patterns are presented as preliminary signals that require confirmation in appropriately controlled and statistically powered studies with predefined endpoints, multiplicity control, longitudinal sampling, and structural validation.

Furthermore, reliance on serum cytokine measurements limits conclusions about local tissue-level immune processes, as cytokine expression in joints, entheses, and axial structures may differ substantially from circulating profiles.

The early loss of non-immunized TLR-KO animals also raises the possibility of survival/selection bias. If mortality was related to genotype-specific vulnerability or unrecognized intercurrent illness, the remaining KO animals available for immunization and follow-up could represent a selected subset, potentially biasing cytokine and terminal serum-marker distributions. Because mortality timing and causes could not be conclusively adjudicated from the available records, and because a contemporaneous non-immunized KO comparator set is absent, we cannot determine whether mortality was genotype- or treatment-related. Accordingly, TLR-related findings should be interpreted as exploratory observations under immunized conditions and require confirmation in balanced cohorts with documented survival and predefined humane endpoints.

Finally, sex effects on immune responses and bone metabolism are well described; because this pilot study was not powered for sex-stratified analyses (and sex was not modeled as a covariate), residual confounding and limited generalizability across sexes cannot be excluded.

Taken together, this pilot study allows several hypotheses for future investigations: (a) Th1/Th2 immune background (C57BL/6J vs. BALB/c) substantially alters the magnitude and temporal kinetics of cytokine responses following proteoglycan immunization; (b) TLR2 and TLR3 signaling are required to sustain Th1/Th17-associated cytokine patterns in this model, whereas TLR deficiency shifts the profile toward increased Th2-type mediators; (c) eosinophil-associated chemokines/cytokines (e.g., IL-5, IL-27, RANTES, and IP-10) represent strain- and TLR-dependent signatures and may be mechanistically linked to distinct inflammatory endotypes in SpA; (d) macrophage-related cytokines/chemokines (e.g., IL-1β, IL-6, MIP-1α/β, MIP-2, and MCP-1/3) are modulated by TLR2/TLR3 status and may serve as candidate biomarkers of inflammatory activity in experimental SpA. A further limitation is that no clinical-like disease activity or severity measures (e.g., arthritis scores/activity indices) and no standardized imaging- or histology-based inflammation severity endpoints were collected as paired outcomes for correlation with the longitudinal cytokine profiles. Therefore, we cannot assess whether the observed cytokine patterns track with disease activity, inflammatory burden, or structural change. Future studies should integrate longitudinal cytokine profiling with predefined clinical scoring and structural/histological endpoints to enable correlation analyses and translational interpretation.

Importantly, this work represents an exploratory screening of candidate cytokine and bone-related readouts in a murine proteoglycan-induced SpA model. Because we did not correlate these readouts with standardized measures of disease severity, structural progression, or functional outcomes, and because the study is not designed to establish diagnostic performance, the findings should not be interpreted as clinically validated biomarkers or as informing therapeutic decision-making. Instead, the results should be viewed as hypothesis-generating patterns that prioritize candidate pathways and readouts for future confirmatory studies incorporating longitudinal clinical/structural endpoints and independent validation.

## 4. Materials and Methods

An established murine model of proteoglycan-induced SpA was used to compare cytokine profiles in Th1- and Th2-prone mice, with special emphasis on the TLR2-, TLR3-, and TLR4-pathways in the Th1-prone model.

### 4.1. Mouse Models for Spondyloarthritis

Imaging analyses showed differences among genotypes: TLR4-KO mice exhibited increased variability in trabecular distribution, a sign of less stable bone structure, whereas TLR-KO mice showed lower variability in trabecular thickness, suggesting enhanced uniformity and robustness [45]. Lower bone volume fractions in the sacrum compared to lumbar vertebrae across genotypes were consistent with human observations of reduced sacral bone volume in SpA. Two different mouse strains, known for their distinct immune response polarizations, were selected to evaluate cytokine expression patterns during experimental SpA. The Th1-prone C57BL/6J strain and the Th2-prone BALB/c strain were chosen to reflect the possible variance in human immune responses to SpA triggers. To explore the influence of innate immune receptors on cytokine profiles in SpA, TLR2-, TLR3-, and TLR4-KO mice bred on the C57BL/6J background were chosen.

**Figure 6:**
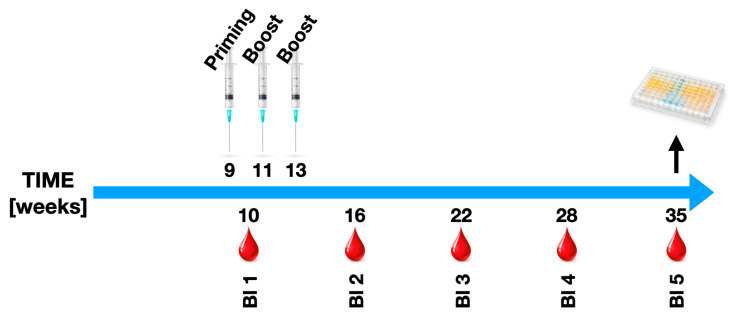
Experimental timeline. Schematic overview of the proteoglycan-induced spondyloarthritis model showing priming immunization (PG; week 9), two booster injections (weeks 11 and 13), longitudinal blood sampling time points (Bl 1–5; weeks 10, 16, 22, 28, and 35), and terminal serum collection for ELISA-based bone-marker analyses (DKK1, Wnt3a, and Noggin). The timeline clarifies the temporal relationship between immunization events, sampling, and terminal endpoint measurements.

In brief, SpA induction was performed by immunizing animals with 100 µg of human fetal cartilage proteoglycan emulsified in Freund’s adjuvant. The first immunization was performed using complete Freund’s adjuvant (CFA) to ensure a strong initial immune response. All animal experiments were approved by the Austrian Ministry of Science, Research, and Economy under the license BMWFW-66011/0111-WF/V3b/2016. Mice were maintained under identical conditions in the animal facility of the Medical University of Innsbruck and provided food and water ad libitum. This was followed by two booster injections with incomplete Freund’s adjuvant (IFA), given at two-week intervals to sustain the immune activation. Immunizations were performed subcutaneously in the flanks of the mice. This approach was chosen as it leads to an inflammatory response with features mimicking key elements of human SpA pathology, including peripheral and axial joint inflammation and potential bone-related changes [26]. Blood samples were collected longitudinally at five predefined time points throughout the experiment. Mice were immunized using a proteoglycan (PG) priming followed by two booster injections at 2-week intervals. Blood samples (B1–B5) were collected at predefined time points spanning the immunization and post-boost observation period, as detailed in the schematic timeline (Figure 6). Terminal blood collection for ELISA-based bone-marker analyses (DKK1, Wnt3a, and Noggin) was performed at the study endpoint. Blood was collected via submandibular venous puncture, enabling repeated sampling in living animals without requiring terminal procedures. The total number of animals per group is provided in Table 1. Efforts were made to minimize animal suffering, and the number of animals used was kept as low as possible while ensuring statistical relevance of the data. However, non-immunized TLR-KO mice were excluded from detailed analysis due to early animal losses, and group sizes were insufficient for robust statistical comparisons.

Mortality in non-immunized KO controls. Non-immunized TLR knockout control cohorts were initially planned but were not available for analysis due to early animal loss during the observation period. Mortality occurred before the planned longitudinal bleeding schedule, thereby precluding the collection of baseline-to-follow-up cytokine trajectories in these KO controls. The available husbandry/monitoring documentation did not allow definitive attribution of the deaths to genotype-related susceptibility versus intercurrent causes. Consequently, KO results are presented as comparisons under immunized conditions only and interpreted as exploratory. Sex composition for each group is provided in Table 1. Given the pilot design and limited group sizes, analyses were not stratified by sex, and sex was not included as a covariate; therefore, sex-related effects could not be assessed.

### 4.2. Quantification of Cytokines and Other Biomarkers

The analysis of cytokines and chemokines in the serum samples was performed using a multiplex bead-based immunoassay, specifically the Mouse ProcartaPlex™ Cytokine & Chemokine 26-Plex Panel provided by ThermoFisher Scientific (Waltham, MA, USA) (Catalog number: EPX260-26088-901). This high-throughput platform enabled simultaneous quantification of 26 biomarkers spanning a broad spectrum of immune responses. The panel included markers relevant to Th1, Th2, and Th17 pathways, chemokines, and growth factors involved in macrophage activation, eosinophil recruitment, and neutrophil chemotaxis. Among these, critical cytokines like TNF-α, IFN-γ, IL-17A, IL-22, IL-23, IL-6, IL-1β, and IL-18 were analyzed, along with chemokines like MCP-1, MIP-1α, MIP-1β, and RANTES.

In addition to cytokine profiling, bone metabolism and formation markers were analyzed with samples from the last bleeding (5th time point). These markers included DKK1, a well-known inhibitor of the Wnt pathway and thus of osteoblast-mediated bone formation, Noggin, an antagonist of bone morphogenetic proteins (BMPs), and Wnt3a, a key ligand of the canonical Wnt signaling pathway. Measurements of these bone-related proteins were carried out using ELISA according to the manufacturers’ protocols (DKK1: R&D Systems (Minneapolis, MN, USA), MKK100; Noggin: Abbexa (Cambridge, UK), abx156251; Wnt3a: Abbexa (Cambridge, UK abx255085). The results were expressed as concentrations in pg/mL for DKK1 and ng/mL for Noggin and Wnt3a.

### 4.3. Statistical Considerations

The statistical analyses were performed using IBM SPSS Statistics 24 (IBM Corporation, Armonk, NY, USA). Due to the exploratory character of this study and the relatively small sample sizes in some groups, non-parametric testing was employed. Specifically, the Mann–Whitney U test was used to compare cytokine levels between groups, including immunized versus non-immunized mice and wild-type versus TLR-deficient animals. Given the non-normal distribution of the data, as confirmed by Shapiro–Wilk tests, non-parametric methods were deemed appropriate. A *p*-value below 0.05 was considered statistically significant. To address multiplicity arising from testing multiple cytokines across multiple time points, we applied false discovery rate (FDR) control using the Benjamini–Hochberg procedure within predefined test families. Families were defined as all cytokines tested for a given pairwise group comparison at a given time point; terminal bone-marker comparisons were treated as a separate family across the bone markers. We report both raw *p*-values and FDR-adjusted *q*-values, and full results are provided in the Supplementary Tables S1–S3. To leverage the repeated-measures design, we additionally evaluated longitudinal kinetics using repeated-measures models including fixed effects for group, time, and the group × time interaction. Because cytokine distributions were right-skewed and included zero values, concentrations were analyzed on a log(1 + x) scale. For each two-group comparison, a random-intercept model (mouse ID as the subject term) was fitted. When mixed-model estimation was numerically unstable for a given analyte/comparison, a generalized estimating equation (GEE) model with an exchangeable within-subject correlation structure was used as a robust alternative. Group × time interaction *p*-values are reported together with family-wise Benjamini–Hochberg FDR *q*-values (across cytokines per comparison). As an effect-size summary of kinetic differences, we calculated per-mouse longitudinal area under the curve (AUC) and report standardized mean differences between groups. Full longitudinal model outputs are provided in the Supplementary Materials (Tables S1–S3).

In accordance with best practices for exploratory analyses, effect sizes were calculated for prespecified key comparisons. For prespecified key comparisons, non-parametric effect sizes (rank-biserial r/Cliff’s δ for pairwise tests and Cohen’s d for longitudinal AUC summaries) were reported to emphasize direction and magnitude; 95% confidence intervals were estimated by bootstrap resampling of the underlying raw data and are provided where statistically identifiable, with complete distributions retained to allow reconstruction of confidence intervals where not explicitly tabulated. Interpretation focuses on the direction and magnitude of effects, while *p*-values and FDR-adjusted *q*-values are provided for completeness (see Supplementary Tables S1–S3 for effect-size reporting).

Radar plots were used to visualize longitudinal data across the five bleeding time points, depicting median cytokine concentrations over time. Data from the 1st bleeding are shown at the top of the radar plots, with subsequent data arranged clockwise. Boxplots were generated to highlight interquartile ranges and median values for endpoint comparisons of the ELISA-based measurements of DKK1, Wnt3a, and Noggin. No correction for multiple comparisons was applied, in line with the pilot nature of this biomarker screening study. For interpretability, in addition to radar plots, key cytokines were summarized using line plots (median with interquartile range) and per-mouse AUC summaries. Minimal detectable effects and power considerations for the available group sizes are summarized in Appendix A.

### 4.4. Ethical Considerations

All experiments involving animals were conducted in strict accordance with ethical standards for animal welfare and were approved by both the local authorities and the Austrian competent authority for animal experimentation under the protocol BMWFW-66011/0111-WF/V3b/2016. Efforts were made to minimize animal suffering, and the number of animals used was kept as low as possible while ensuring statistical relevance of the data.

## 5. Conclusions

This pilot study using a proteoglycan-induced spondyloarthritis mouse model identified exploratory differences in cytokine patterns between Th1-prone C57BL/6J and Th2-prone BALB/c strains, and further observed altered cytokine distributions and terminal bone-marker levels (DKK1, Wnt3a, Noggin) in proteoglycan-immunized TLR2/3/4-deficient mice on the C57BL/6J background. However, the evidence remains preliminary due to small, imbalanced groups, the absence of non-immunized knockout controls, the lack of corresponding knockout controls on the BALB/c background, and the exploratory nature of the statistical testing without multiplicity adjustment. Moreover, bone-related serum markers were measured only at the terminal time point and without structural endpoints within this study (e.g., imaging or histology), and no clinical validation or external replication was performed. Therefore, the findings should be interpreted as hypothesis-generating and not as establishing stable biomarker profiles or therapeutic targets. Overall, these data define hypothesis-generating cytokine and bone marker patterns associated with immune background and TLR signaling in a proteoglycan-induced murine SpA model; however, they do not establish validated biomarkers or therapeutic implications and must be confirmed in independent, adequately powered studies that incorporate clinical, functional, and structural outcome measures. Taken together, this pilot study highlights model- and strain-dependent serum cytokine patterns in a proteoglycan-induced SpA mouse model, underscoring the importance of immune background and temporal context. These findings are hypothesis-generating and require validation using longitudinal, tissue-level, and clinically anchored endpoints.

## Figures and Tables

**Table 1:** Overview of mouse models used for experimental SpA and cytokine profiling. C57BL/6J mice were used as a Th1-prone strain and BALB/c mice as a Th2-prone strain. TLR-deficient (TLR-KO) mice enabled targeted assessment of TLR2, TLR3, and TLR4 pathways in regulating inflammatory and bone-related biomarkers. Mouse sourcing: BALB/c WT mice were obtained from Charles River (Schweinfurt, Germany); TLR2-KO and TLR4-KO mice were obtained from Oriental BioServices Inc. (Kyoto, Japan). Sex composition (male/female) for each group is reported in Table 1. WT, wild-type.

Strain	Genotype	Immunization	Size (*n*)	Sex (M/F)	Purpose of Inclusion
BALB/c	WT	No	7	7/0	Th2-prone control group as baseline reference
BALB/c	WT	Yes *	9	9/0	Th2-prone model of induced SpA
C57BL/6J	WT	No	8	8/0	Th1-prone control group as baseline reference
C57BL/6J	WT	Yes *	16	16/0	Th1-prone model of induced SpA
C57BL/6J	TLR2-KO	Yes *	7	7/0	Th1-prone TLR2-KO mice to study role of TLR2
C57BL/6J	TLR3-KO	Yes *	8	8/0	Th1-prone TLR3-KO mice to study role of TLR3
C57BL/6J	TLR4-KO	Yes *	3	3/0	Th1-prone TLR4-KO mice to study role of TLR4

* immunization with human cartilage proteoglycan emulsified with complete (1st immunization) and incomplete Freund’s adjuvant (2nd and 3rd immunization).

## Data Availability

The raw data supporting the conclusions of this article will be made available by the authors on reasonable request.

## Supplementary Materials

The following supporting information can be downloaded at: https://www.mdpi.com/article/10.3390/ijms27031337/s1.

## Abbreviations

The following abbreviations are used in this manuscript:

AS	Ankylosing Spondylitis
AUC	Area Under the-Curve
BALB/c	Bagg Albino Laboratory-Bred Strain c (Th2-prone mouse strain)
BMP	Bone Morphogenetic Protein
C57BL/6J	Inbred C57 Black 6J Mouse Strain (Th1-prone)
CFA/IFA	Complete/Incomplete Freund’s Adjuvant
CRP	C-Reactive Protein
CXCL1/Gro-α	Chemokine (C-X-C motif) Ligand 1/Growth-Regulated Oncogene Alpha
DKK-1	Dickkopf-related Protein 1
ELISA	Enzyme-Linked Immunosorbent Assay
ER	Endoplasmic Reticulum
ERAP1/2	Endoplasmic Reticulum Aminopeptidase 1 and 2
ESR	Erythrocyte Sedimentation Rate
FDR	False Discovery Rate
GEE	Generalized Estimating Equation
HLA-B27	Human Leukocyte Antigen B27
HR-pQCT	High-resolution peripheral quantitative computed tomography
IFA	Incomplete Freund’s Adjuvant
IFNγ	Interferon Gamma
IL	Interleukin
IL-23R	Interleukin-23 Receptor
IP-10	Interferon Gamma-Induced Protein 10 (CXCL10)
KO	Knockout
MCP-1/3	Monocyte Chemoattractant Protein 1 and 3
MHC	Major Histocompatibility Complex
MIP-1α/β/2	Macrophage Inflammatory Protein 1 alpha, 1 beta, and 2
MΦ	Macrophage
NK	Natural Killer (cell)
pg/mL	Picogram per Milliliter
pQCT	Peripheral Quantitative Computed Tomography
RANTES	Regulated upon Activation, Normal T Cell Expressed and Secreted (CCL5)
RORγt	Retinoic Acid Receptor-Related Orphan Receptor Gamma t
SEM	Standard Error of the Mean
SpA	Spondyloarthritis
STAT3	Signal Transducer and Activator of Transcription 3
TGF-β	Transforming Growth Factor Beta
Th1/Th2/Th17	T-helper Cell Type 1, 2, and 17
TLR	Toll-like Receptor
TNF-α	Tumor Necrosis Factor Alpha
UPR	Unfolded Protein Response
WT	Wild-Type
Wnt3a	Wingless-Type MMTV Integration Site Family Member 3A

## Appendix A. Power Considerations (Minimal Detectable Effects)

Given the markedly unbalanced group sizes, statistical power is limited, and only comparatively large effects can be detected reliably. To provide a transparent benchmark, we estimated the minimal detectable standardized mean difference (Cohen’s *d*) for representative two-group comparisons under a two-sided α = 0.05 and 80% power (approximate; assumes independent samples and does not account for repeated-measures structure or multiple testing). Under these assumptions, the minimal detectable *d* is approximately:

- C57BL/6J WT immunized (*n* = 16) vs. BALB/c WT immunized (*n* = 9): *d* ≈ 1.22
- TLR2-KO (*n* = 7) vs. C57BL/6J WT immunized (*n* = 16): *d* ≈ 1.33
- TLR3-KO (*n* = 8) vs. C57BL/6J WT immunized (*n* = 16): *d* ≈ 1.27
- TLR4-KO (*n* = 3) vs. C57BL/6J WT immunized (*n* = 16): *d* ≈ 1.87

These benchmarks indicate that the study is primarily sensitive to significant effects, and that inference for the TLR4-KO subgroup (*n* = 3) is not statistically stable. Therefore, TLR4-KO findings are reported descriptively and are not interpreted as evidence of an effect. Future confirmatory studies should predefine primary endpoints, ensure balanced groups (including non-immunized KO controls), and apply multiplicity control to support robust inference.
